# Novel Anti-Cancer Stem Cell Compounds: A Comprehensive Review

**DOI:** 10.3390/pharmaceutics16081024

**Published:** 2024-08-01

**Authors:** Shanchun Guo, Shilong Zheng, Mingli Liu, Guangdi Wang

**Affiliations:** 1 RCMI Cancer Research Center and Department of Chemistry, Xavier University of Louisiana, New Orleans, LA 70125, USA; szheng@xula.edu; 2 Department of Biochemistry & Immunology, Morehouse School of Medicine, Atlanta, GA 30310, USA; mliu@msm.edu

**Keywords:** targeted therapy, Notch, Wnt/β-catenin, ALDH, compound

## Abstract

Cancer stem cells (CSCs) possess a significant ability to renew themselves, which gives them a strong capacity to form tumors and expand to encompass additional body areas. In addition, they possess inherent resistance to chemotherapy and radiation therapies used to treat many forms of cancer. Scientists have focused on investigating the signaling pathways that are highly linked to the ability of CSCs to renew themselves and maintain their stem cell properties. The pathways encompassed are Notch, Wnt/β-catenin, hedgehog, STAT3, NF-κB, PI-3K/Akt/mTOR, sirtuin, ALDH, MDM2, and ROS. Recent studies indicate that directing efforts towards CSC cells is essential in eradicating the overall cancer cell population and reducing the likelihood of tumor metastasis. As our comprehension of the mechanisms that stimulate CSC activity, growth, and resistance to chemotherapy advances, the discovery of therapeutic drugs specifically targeting CSCs, such as small-molecule compounds, holds the potential to revolutionize cancer therapy. This review article examines and analyzes the novel anti-CSC compounds that have demonstrated effective and selective targeting of pathways associated with the renewal and stemness of CSCs. We also discussed their special drug metabolism and absorption mechanisms. CSCs have been the subject of much study in cancer biology. As a possible treatment for malignancies, small-molecule drugs that target CSCs are gaining more and more attention. This article provides a comprehensive review of the current state of key small-molecule compounds, summarizes their recent developments, and anticipates the future discovery of even more potent and targeted compounds, opening up new avenues for cancer treatment.

## 1. Introduction

Stem cells (SCs) that are found in niches possess the ability to undergo self-renewal and specialize into many cell types within adult somatic tissues. Cancer stem cells (CSCs) are a distinct group of cells having stem cell characteristics that are present within malignancies. They have a vital function in initiating and facilitating the growth and advancement of cancer. This is achieved by disrupting various signaling pathways, leading to cellular and tumor-specific molecular diversity. As a result, CSCs are regarded as the highest point in the hierarchical model of tumorigenesis, progression, metastasis, and resistance to drugs [1,2,3]. Several malignancies have been found to exhibit an enhanced ability to initiate tumor growth, partially replicate the diversity of cells and molecules, and overall display greater resistance to traditional anticancer treatments compared to the responses of other tumor cells. Previous research has discovered CSCs that have a significant impact on the start, development, dissemination, and resistance to specific therapies of solid tumors [4,5,6,7].

SCs and CSCs share functional and phenotypic similarities, including the capacity for self-renewal and differentiation. Nevertheless, there exist notable distinctions in the biological activities of SCs and CSCs, mostly ascribed to a severe disturbance in the capacity of CSCs to renew themselves without assistance. Contrary to SCs, which undergo differentiation and ultimately produce specialized offspring, CSCs generate offspring that display unregulated proliferation and do not undergo terminal differentiation. CSCs acquire a malignant phenotype due to variations in their cell-cycle characteristics, division, replicative potential, molecular pathway activation and inactivation, and DNA damage control. At present, the clinical difficulties that persist are tumor recurrence, metastasis [7,8]. Since CSCs have distinct metabolic properties, a new approach is the targeting of CSCs [9,10]. CSCs exhibit resistance to traditional cancer treatments and typically possess a high capacity for tumor formation and metastasis [11]. CSCs also exhibit stemness acquired through the process of epithelial-to-mesenchymal transition (EMT), which refers to their capacity for self-renewal and differentiation [12,13]. The concept of stemness plays a crucial role in driving the development and progression of cancer. It allows CSCs to undergo self-renewal, invade surrounding tissues, spread to distant sites, and regenerate tumors [11,14,15]. 

**Figure 1:**
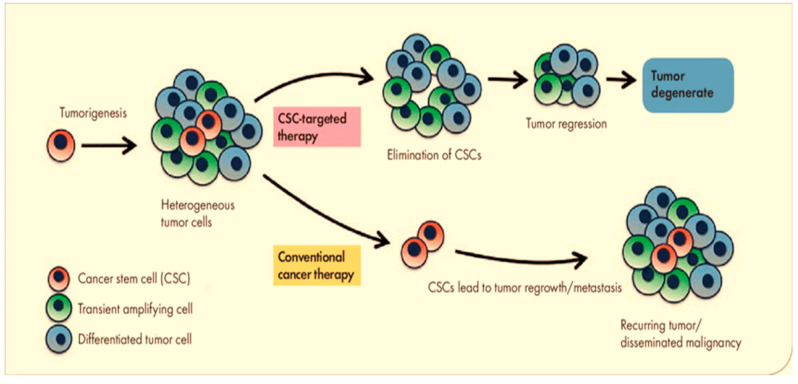
In the tumor CSC model, only CSCs have tumor-initiating capability.

All cells exhibit the same tumorigenic activity when seen through the lens of the tumor clonal evolution model [16]. CSCs, on the other hand, are the only cells that exhibit self-renewal capacity, tumor-initiating capability, and pluripotency in the tumor CSC model demonstrated in Figure 1 [9,17]. This may explain why early tumor shrinkage for evaluating the effects of therapies is usually poorly predictive of the patient outcome and overall survival [18,19,20]. Despite the fact that standard chemotherapy may be able to eliminate the majority of cancer cells that are not stem cells, CSCs are more resistant to chemotherapy and have the potential to cause tumor relapse [12,21]. Non-stem cancer cells can acquire cancer stemness when they undergo dedifferentiation during traditional cancer therapy through the connections between EMT and differentiation status [22,23,24].

Increasing research suggests that specifically focusing on CSCs is essential for eradicating the entire population of cancer cells. As our comprehension of the mechanisms that drive CSC activity, progression, and chemoresistance improves, the creation of therapeutic drugs or treatment methods specifically targeting CSCs may result in significant advancements in malignancy therapy [21,25,26]. For the purpose of our investigation, we have focused our attention on the small-molecule compounds that target certain pathways that are closely connected with the renewal and stemness of CSCs.

## 2. Small-Molecular Compounds of Cancer Stem Cells Related to Specific Signaling Pathways 

### 2.1. Small-Molecule Compounds Targeting Notch Signaling Pathway

Notch signaling performs the role of receptors for ligands that are bound to membranes. Notch1 through Notch4 are the four Notch genes that it possesses, and it possesses five ligands: Delta-like 1 (DLL1), DLL3, DLL4, and Jagged1, Jagged2 [27,28]. Notch receptors have an intracellular domain and an extracellular domain, which have several epidermal growth factor (EGF) domains [29]. The Notch proteins may impact many types of cell activities, including apoptosis, angiogenesis, differentiation, and proliferation [30,31]. Therefore, Notch influences morphogenesis and organogenesis [32]. Notch receptor activation is induced by Jagged or Delta ligands in neighboring cells.

The fundamental roles of Notch signaling are implicated in developmental processes, during the regulation of embryonic development and adult tissue homeostasis; during the differentiation, maintenance, and cellular homeostasis of SCs [33,34]. There is a correlation between CSCs and Notch receptors and/or ligands in a number of different types of tumors [27,31,35,36,37]. Notch activity and epigenetic modulation of the Notch regulators have been described in breast CSCs (BCSCs), which are cells that originate from breast cancer [38,39,40,41]. The activation of Notch1 improves survival of CD24(+) CD29(high) progenitor cells via a cyclin D1-dependent route, hinders self-renewal of mammary SCs, and makes their transformation easier [42]. The HER2 has Notch-RBP-Jκ binding sites in its promoter [43] and is triggered in both types of breast stem cells [39,44] and BCSCs by Notch1 signaling. The presence of the erythropoietin receptor (EpoR) was observed on the surface of BCSCs, and it was shown that erythropoietin (Epo) interacts with Notch1 to sustain the ability of BCSCs to self-renew [45]. The activation of Notch results in the creation of dimers of Hes and/or Hey proteins, which inhibit the transcription of several genes by capturing transcriptional activators or associating with co-repressors [32,46,47]. In recent years, BCSCs were demonstrated to be correlated with several oncogenes, such as cyclo-oxygenase (COX)-2 [48], HER2 [49,50], MYC[51], and Akt [52], in addition to transcription factors like STAT3 [53], HIF-1 [54], and NF-*κ*B [55]. As Notch signaling interacts with these oncogenes and/or transcriptional factors [31], through these signals it may have an effect on BCSCs and the progression of breast cancer. Table 1 displays specific small chemical compounds that target cancer stem cells across various pathways, including the Notch pathway.

**Figure 2:**
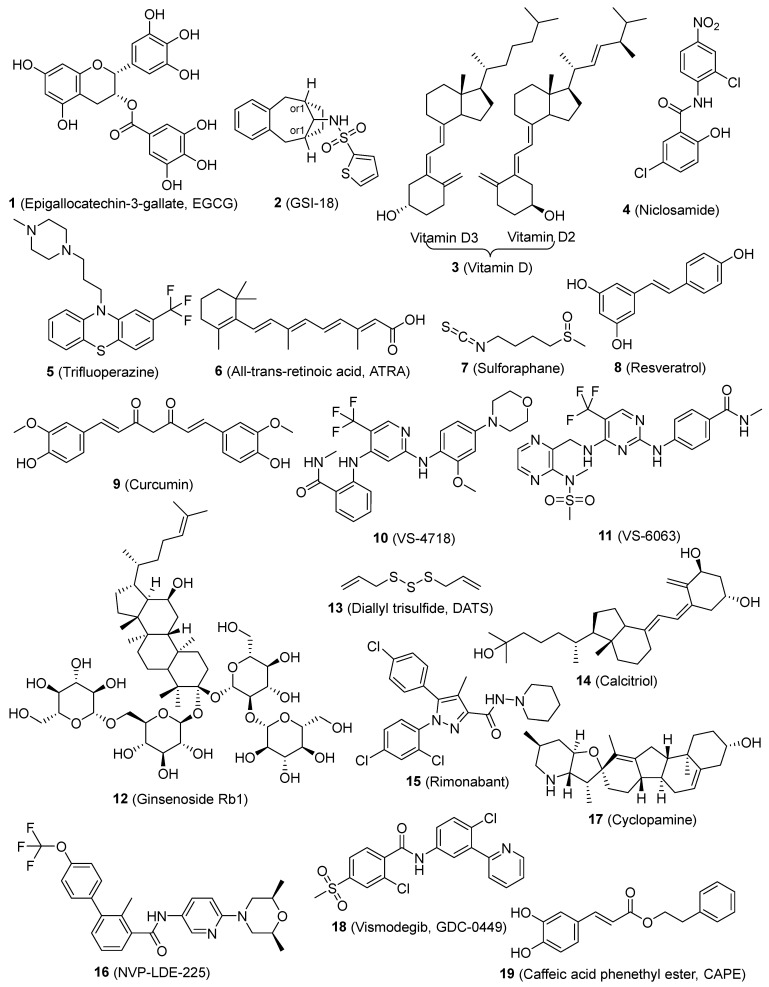
Chemical structures of targeted small-molecule compounds of cancer stem cells.

Epigallocatechin-3-gallate (EGCG) (**1**, Figure 2), which is the primary polyphenol found in green tea, effectively prevents the self-renewal ability of head-neck squamous carcinoma (HNSC) CSCs. The primary site of absorption for EGCG is the intestine, and the metabolism of EGCG by gut microbes is crucial before it can be absorbed [119]. EGCG, as a means of suppressing HNSC CSC characteristics, reduced the transcriptional activity of Notch, hence inhibiting Notch signaling [56]. Stem-like cells found in brain tumors appear to be specifically susceptible to GSI-18 (**2**, Figure 2), which inhibits the Notch pathway [59]. The vitamin D compounds (**3**, Figure 2) also decreased the expression of Notch signaling molecules, such as Notch1, Notch2, Notch3, JAG1, JAG2, HES1, and NFκB, which play a role in maintaining BCSCs. Vitamin D has the potential to be utilized as an agent for preventing triple-negative breast cancer (TNBC) by controlling CSCs and promoting differentiation [60]. Niclosamide (**4**, Figure 2) is a well-established antihelminthic medication that disrupts the functioning of mitochondria in intestinal parasites. Studies have shown that **4** has the potential to be an effective anticancer drug [120]. However, its limited ability to dissolve in water must be addressed before additional preclinical and clinical research can be performed. **4** was discovered to be a CSC inhibitor using a dye exclusion technique. It suppressed the activity of Notch, Wnt, and SHH stem pathways, hindered the development of spheroids, and triggered programmed cell death in the side population sphere of breast cancer. Niclosamide’s therapeutic properties were also validated through animal experiments [61].

### 2.2. Small-Molecule Compounds Targeting Wnt/β-Catenin Signaling Pathway

The Wnt/β-catenin signaling pathway is crucial in controlling various biological processes, including cell proliferation, angiogenesis, polarity, asymmetric cell division, tissue homeostasis, and cancer development [121,122,123]. The Wnt/β-catenin signaling system has the ability to stimulate the formation of pluripotent stem (iPS) cells, hence enhancing the process of reprogramming somatic cells. This highlights the significance of this pathway in promoting the renewal and multipotency of stem cells [124,125,126]. Abnormal Wnt/β-catenin signaling has been widely documented in several types of cancers [127,128,129,130]. The importance of β-catenin in maintaining the characteristics of epidermal CSCs was recognized early on. Removing this gene leads to the elimination of epidermal CSCs [131]. Several publications have demonstrated that intestinal SCs can undergo a transformation into CSCs due to abnormal Wnt/β-catenin signaling. This suggests that SCs are the cells responsible for initiating tumors in the intestine and highlights the significance of Wnt/β-catenin in facilitating the transition from SCs to CSCs during the development of intestinal cancer [132,133,134]. Mammary SCs were also identified as target cells in the process of Wnt-induced mammary gland tumorigenesis [135,136,137]. Wnt signaling can impact distinct pools of mammary SCs, giving rise to different types of tumors [138]. A comprehensive review has been conducted on the small-molecule compounds of Wnt [139]. Here, we discuss a few Wnt inhibitors that were reported to target CSCs.

Trifluoperazine (**5**, Figure 2) hinders the production of CSC tumor spheroids and reduces the expression of the CSC markers (CD44/CD133). **5** hinders the Wnt/β-catenin signaling pathway in lung cancer sphere that is resistant to gefitinib. The coadministration of **5** with either gefitinib or cisplatin effectively circumvents drug resistance in lung CSCs. **5** suppresses the growth of tumors and increases the inhibitory effect of gefitinib in animal models of lung cancer metastasis and orthotopic CSCs [62]. All-trans retinoic acid (ATRA) (**6**, Figure 2) is commonly used to treat a range of disorders characterized by excessive cell growth and inflammation. Nevertheless, the therapeutic effectiveness of the substance is at risk because of its limited solubility and durability [140]. The latter was overcome by incorporating it into a solid matrix of lipidic nanoparticles (SLNs). ATRA repressed the expression of the stem cell markers Oct4, Sox2, Nestin, and CD44 and hindered the growth of HNSC, thyroid, and breast CSCs both in laboratory conditions and in living organisms. The potential anti-tumor effects of ATRA may be linked to the suppression of Wnt/β-catenin signaling [63,64,65]. Sulforaphane (SFN) (**7**, Figure 2), a prominent member of the isothiocyanates, is found in abundance in cruciferous plants and is renowned for its remarkable anti-cancer properties [141]. SFN suppresses breast CSCs and reduces the activity of the Wnt/β-catenin pathway, which is responsible for the self-renewal of these cells. SFN has the potential to act as a chemopreventive drug for breast CSCs and should be further evaluated in clinical settings [66]. Resveratrol (**8**, Figure 2), a naturally occurring polyphenolic molecule, hinders the growth of breast CSCs and triggers a process called autophagy by suppressing the Wnt/β-catenin signaling pathway [67]. A natural substance called curcumin (**9**, Figure 2) blocks the migration of breast CSCs by intensifying the negative feedback loop between E-cadherin and β-catenin [70]. Additionally, **9** demonstrated its interventional effect on lung CSCs through the suppression of sonic hedgehog and Wnt/β-catenin pathways [71]. EGCG (**1**) efficiently decreases the activity of lung CSCs by suppressing the formation of tumorspheres, reducing the expression of markers specific to lung CSCs, inhibiting cell growth, and promoting apoptosis. EGCG suppresses Wnt/β-catenin activation, while the increase in Wnt/β-catenin counteracts the inhibitory impact of EGCG on lung CSCs [57]. VS-4718 and VS-6063 (**10** and **11**, Figure 2) selectively target CSCs by inhibiting the activity of focal adhesion kinase (FAK). A fascinating interaction between FAK and the Wnt/β-catenin pathway has been discovered, in which FAK inhibition prevents the activation of β-catenin by decreasing the phosphorylation of tyrosine 654 on β-catenin. The selective targeting of CSCs by FAK inhibitors offers a logical basis for the therapeutic advancement of FAK inhibitors with the goal of enhancing long-lasting responses in cancer treatment [73]. The natural product ginsenoside Rb1 (**12**, Figure 2) demonstrates strong cytotoxic effects on CSCs obtained from patients with ovarian carcinoma. **12** further enhances the sensitivity of CSCs to therapeutically significant amounts of cisplatin and paclitaxel. These effects are associated with the Wnt/β-catenin signaling pathway. Furthermore, no signs of toxicity were seen when administering doses of **12** that resulted in an anti-CSC impact [74]. Diallyl trisulfide (DATS) (**13**, Figure 2), a naturally occurring organosulfur chemical found in garlic, effectively reduced breast CSCs by preventing the activation of the Wnt/β-catenin pathway [75]. Calcitriol (**14**, Figure 2) reduces the number of ovarian CSCs by blocking their Wnt signaling pathway, which hinders the growth of xenograft tumors [76]. Rimonabant (**15**, Figure 2) can effectively decrease the growth of both tumor-differentiated cells and colon CSCs by regulating Wnt activity. It also has the ability to manage their survival throughout extended periods of cultivation. **15** exhibits non-toxicity against healthy colonic epithelial cells, indicating its potential specificity towards cancer cells [77].

### 2.3. Small-Molecule Compounds Targeting Hedgehog Signaling

The hedgehog signaling pathway plays a crucial role in embryonic development, tissue regeneration, maintenance of adult tissue balance, and the formation of tumors [142,143,144,145]. Hedgehog family ligands, Desert hedgehog (Dhh), Indian hedgehog (Ihh), and Sonic hedgehog (Shh), can generate mature peptides after autoprocessing and lipid modification [146,147,148]. Tumor initiation and progression can occur as a result of abnormal activation of the hedgehog signaling pathway [145,149]. The hedgehog signaling pathway induces the expression of JAG2, a ligand of the Notch signaling pathway, during the development of cancer [150]. Hedgehog signaling pathway facilitates cellular evasion of apoptosis, disrupts cellular energy metabolism, modulates the process of EMT, aids in evading the immune system, sustains CSCs, and promotes metastasis.

The connection between Notch and hedgehog triggers the creation of Hes3 and Shh by activating several signaling pathways, such as Akt, STAT3, and mTOR. These pathways play a crucial role in promoting the survival of neural stem cells [151]. The presence of abnormal hedgehog signaling in human breast cancer can affect the capacity of BCSC to self-renew and differentiate [152].

Emerging data from laboratory experiments conducted in test tubes and living organisms indicates that the improper reactivation of the Shh signaling pathway controls genes that stimulate the growth of different types of human CSCs. NVP-LDE-225 (**16**, Figure 2) suppresses epithelial–mesenchymal transition (EMT) and development of human prostate CSCs in mice by blocking the Shh signaling system [78]. Curcumin (**9**) has been identified as a promising therapeutic drug and demonstrates cellular-level actions that contribute to its wide range of health benefits [153]. Multiple comprehensive studies have conclusively shown that it has the capacity to augment the therapeutic efficacy of diverse bioactive substances. The documented therapeutic benefits of this substance are numerous, mainly due to its antioxidant and anti-inflammatory qualities. However, its effectiveness is limited by low bioavailability caused by insufficient absorption, quick metabolism, and excretion. **9** suppressed the functions of bladder CSCs by blocking the SHH pathway. This suggests that **9** could be a promising chemopreventive therapy for treating bladder cancer [72]. Cyclopamine (**17**, Figure 2) induced a significant decrease in the growth of adherent glioma lines that predominantly express Gli1, a crucial target of the hedgehog pathway. However, this reduction was not observed in glioma lines that showed no signs of pathway activity. **17** effectively decreased or eradicated the population of stem-like cells in gliomas [79]. Vismodegib (GDC-0499) (**18**, Figure 2) suppressed the growth of pancreatic CSCs and triggered programmed cell death. The molecular mechanism of **18** involves inhibiting the actions of Smoothened in the SHH signaling pathway. Hence, **18** can be utilized for the treatment of pancreatic cancer by specifically targeting pancreatic CSCs [80].

### 2.4. Small-Molecule Compounds Targeting the NF-κB Signaling Pathway

The NF-κB transcription factors control the expression of several crucial genes involved in cell proliferation and survival, as well as innate and adaptive immunity. NF-κB played a role in promoting tumor inflammation [154] and the angiogenesis of tumors [155], which is triggered in a diverse range of cancers [154,156,157]. The NF-κB family comprises RelA (p65), RelB, c-Rel, p105/p50, and p100/p52. These molecules can form hetero- or homodimers and are a collection of evolutionarily conserved molecules. The most prevalent type, known as the canonical route, is the p65/p50 heterodimer [156,158]. NF-κB is triggered by various stimuli, including pro-inflammatory cytokines, interleukin-1β (IL-1β), bacteria and lipopolysaccharides (LPS), EGF, T- and B-cell mitogens, viral proteins, physical and chemical stresses, and double-stranded RNA [157,159]. NF-κB is also activated by cellular stresses, such as chemotherapeutic drugs and ionizing radiation [160]. The activation of NF-κB was found to be necessary in the neoplastic transformation induced by arsenite, which exhibits EMT and CSC-like characteristics in human keratinocytes [161], human ovarian CSC metastatic property [162], and human cervical CSC growth [82], while preventing differentiated glioma CSCs from developing a fully-fledged postmitotic phenotype [163]. In addition, studies also indicate that NF-*κ*B regulates the self-renewal of breast CSCs in a model of HER2-dependent tumorigenesis [164].

**Figure 3:**
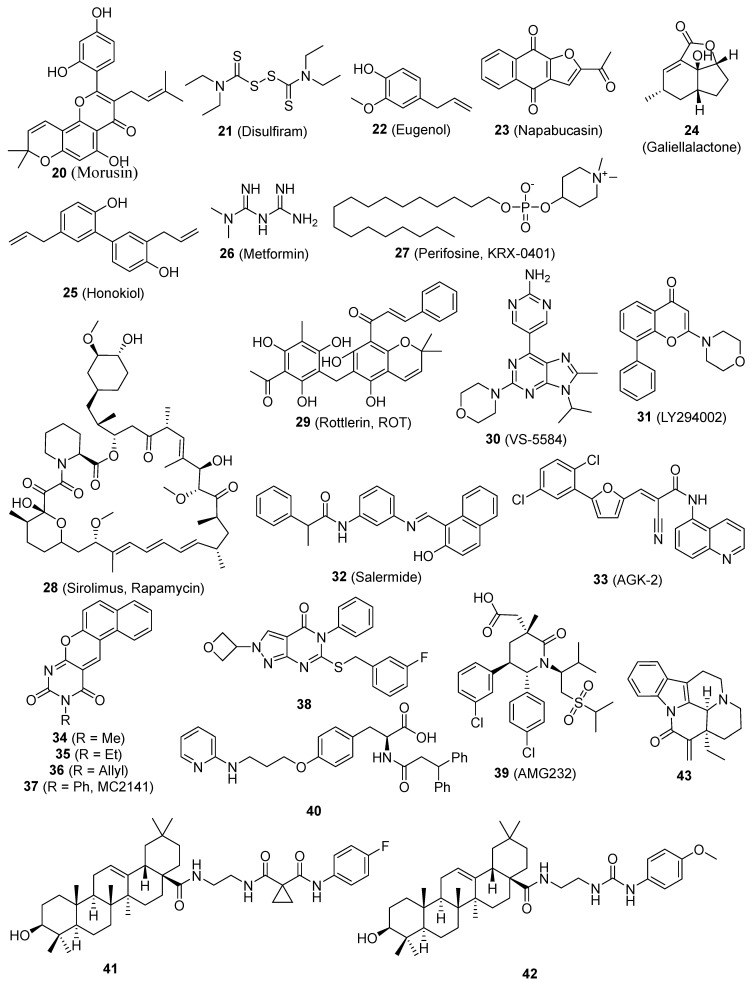
Chemical structures of targeted small-molecule compounds of cancer stem cells.

Caffeic acid phenethyl ester (CAPE) (**19**, Figure 2), specifically caffeic acid phenethyl ester, hinders the proliferation of MDA-MB-231 (MDA-231) cells, suppresses the expression of the mdr gene, and inhibits NF-κB, EGFR, and VEGF. The administration of CAPE resulted in a dose-dependent suppression of self-renewal in breast CSCs and the generation of progenitor cells. The administration of CAPE results in considerable alterations in the features of breast CSCs. These changes include the suppression of self-renewal, the generation of progenitor cells, and the growth of clones in soft agar. Additionally, there is a notable decrease in the content of CD44, which is indicative of a reduced potential for malignancy [81]. Morusin (**20**, Figure 3) is a compound found in mulberry, specifically in the branch and root bark of several Morus species. It is a prenylated flavone and has a wide range of pharmacological effects. Nevertheless, there is a dearth of comprehensive research regarding its absorption and disposal [165]. **20** exhibits anti-cancer properties via reducing the activity of NF-κB, a protein that is increased in CSCs. **20** possesses the capacity to selectively target and eliminate CSCs and can hinder the growth and movement of human cervical cells by reducing the activity of NF-κB, hence inducing apoptosis [82]. The combination of disulfiram (**21**, Figure 3) and copper suppressed the growth of breast CSCs and increased the effectiveness of paclitaxel in breast cancer cell lines. This effect is likely due to the simultaneous activation of reactive oxygen species (ROS) and inhibition of NF-κB [83]. Eugenol (**22**, Figure 3) enhances the inhibitory effect of cisplatin on the NF-κB signaling pathway. Comparable outcomes were noted regarding the increase in cell division, suppression of the transformation from epithelial to mesenchymal cells, and indicators of cellular pluripotency in tumor xenografts. The findings offer compelling preclinical evidence for the combination of cisplatin and **22** as a therapeutic strategy for TNBC. This method specifically targets the resistant ALDH-positive cells and inhibits the NF-κB pathway [84].

### 2.5. Small-Molecule Compounds Targeting STAT3 Signaling Pathway

In both normal and cancer cells, the transcription factor STAT3 plays an essential role in a number of signal transduction pathways [166]. STAT3 is commonly activated across diverse cellular contexts [167]. STAT3 has a crucial role in governing both regular SCs and CSCs. P63, a constituent of the p53 protein family, exhibits both physical and functional associations with STAT3 and participates in its activities. A recent review thoroughly examined the functions of STAT3 in cancer and CSCs [168]. STAT3 is excessively active in numerous cancer types and has demonstrated to be a significant route in the propagation of cancer mediated by CSCs. Napabucasin (BBI608) (**23**, Figure 3) was taken in, processed into M1 as the only significant metabolite present in the bloodstream, and mainly eliminated through the feces. The administration of a single 240-mg dose orally was typically well tolerated [169]. **23** is a novel cancer stemness inhibitor now undergoing Phase III clinical trials. Clinical trials of **23** have demonstrated promising anti-tumor effects both as a standalone treatment and when used in combination with traditional therapies. Furthermore, **23** does not exhibit any notable pharmacokinetic interactions when used in combination therapy. **23** has the ability to effectively target CSCs, hence suppressing the spread of cancer metastasis and preventing the recurrence of the disease in patients with different forms of cancer [85,86,87]. **23** inhibits the activity of stem cells in cancer cells by specifically targeting STAT3 [8]. Curcumin (**9**) and EGCG (**1**) specifically blocked the process of STAT3 phosphorylation and maintained the link between STAT3 and NF-kB. They act as antitumor medicines that inhibit the growth of breast CSCs [58]. ALDH expression serves as an indicator for the presence of CSC-like cells in many human prostate cancer cell lines. Furthermore, ALDH+ cells demonstrate the presence of phosphorylated STAT3. Treatment with Galiellalactone (**24**, Figure 3) decreases the percentage of prostate cancer cells that express ALDH and triggers apoptosis in these ALDH-positive cells. The results emphasize the potential of targeting the STAT3 pathway in prostate cancer cells, particularly prostate CSC-like cells, as a feasible therapeutic approach. Additionally, **24** shows promise as a molecule for future development of drugs for prostate cancer [88]. Honokiol (HNK) (**25**, Figure 3) suppressed the movement of individual cells and eliminated the characteristics of stem cells in breast cancer cells. Mechanistic investigations demonstrated that HNK hindered the phosphorylation/activation of STAT3 in a manner that relied on LKB1, hence blocking its recruitment to the typical binding sites of the promoters, which include Nanog, Oct4, and Sox2. The molecular investigations of xenografts treated with HNK confirmed the mechanistic findings observed in vitro [89]. Metformin (**26**, Figure 3), the primary medication used to treat diabetes, hinders the process of cell transformation and specifically targets and eliminates CSCs in breast cancer cell lines. **26** selectively hinders the movement of NF-κB into the nucleus and the process of STAT3 phosphorylation in CSCs when compared to non-stem cancer cells within the same group. Metformin has the potential to impede metabolic stress and activate the inflammatory pathways linked to several types of cancer [90].

### 2.6. Small-Molecule Compounds Targeting PI-3K/Akt/mTOR Signaling Pathway

The phosphatidylinositol-3-kinase (PI-3K)/Akt/mTOR pathway plays a pivotal role in regulating cell proliferation, growth, motility, angiogenesis, and survival in tumor cells [170,171]. mTOR is a serine/threonine kinase that frequently acts as a downstream mediator of PI-3K/Akt signaling in tumor cells. Activation of PI-3K/Akt is highly associated with downstream targets, namely mTOR-mediated 4BEP-1 and S6K1 [172]. The MAPK pathway also controls mTOR in the activation of pro-angiogenic/inflammatory chemicals in tumor cells [173]. It is also possible for mTOR to phosphorylate Akt [174]. The abnormal activation of the mTOR pathway is believed to have significant implications for the proliferation of cancer cells and their resistance to anti-cancer drugs across various forms of cancer [175,176,177]. The PI-3K/Akt/mTOR pathway plays a crucial role in the process of EMT during the development of cancer [178]. The occurrence of several changes in cancer is mostly due to heightened activation of the PI3-K/Akt/mTOR pathway [171,179].

Multiple data suggest that the PI-3K/Akt/mTOR signaling pathway is significant in the biology of CSCs. Furthermore, CSCs exhibit greater sensitivity to blockage of this system using small compounds compared to regular SCs [180]. The CD133/PI-3K/Akt signaling axis was discovered to play a crucial role in the behavior of glioma CSCs [181]. Perifosine (KRX-0401) (**27**, Figure 3), an Akt inhibitor, was reported to reduce serial mammosphere formation and may be a novel agent as putative therapy for breast CSCs [52]. Sirolimus (Rapamycin) (**28**, Figure 3), by mTOR inhibition, effectively reduces EMT and CSC-like properties in colorectal cancer cells [91]. Rottlerin (ROT) (**29**, Figure 3), a commonly utilized inhibitor of protein kinase C-delta (PKC-δ), has been found to induce autophagy in human pancreatic CSCs via inhibiting the PI-3K/Akt/mTOR pathway [92]. The deregulation of the PI-3K pathway in numerous types of cancer has firmly established PI-3K as a universally recognized target for treatment. **29** triggered autophagy, which subsequently led to death in human pancreatic CSCs. This outcome was accomplished by suppressing the PI3K/Akt/mTOR pathway and stimulating the caspase cascade. The delivery of **29** led to a decrease in cell viability, which was influenced by the dosage and time of exposure, as well as the development of cytoplasmic vacuoles. **29** can initiate autophagy, a biological process that leads to the demise of pancreatic CSCs [92]. When comparing VS-5584 (**30**, Figure 3) to non-CSC in solid tumor cell populations, it is seen that **30** is considerably more potent, exhibiting a 30-fold enhancement in its capacity to inhibit the proliferation and survival of CSCs. **30** selectively decreases the numbers of CSC in several mice xenograft models of human cancer. Similarly, the ex vivo therapy with **30** specifically decreased the population of CSC in tumors that were surgically removed from patients with breast and ovarian cancer. Research on the mechanisms involved has demonstrated that effectively targeting CSC necessitates the suppression of many elements within the PI-3K-mTOR pathway. Merely silencing a single PI3K isoform or mTOR is insufficient to reproduce the effects of **30** [93]. LY294002 (**31**, Figure 3), which inhibits the PI-3K enzyme, decreases the growth, creation of spherical structures, and ability of colon CSCs to renew themselves. Additionally, it inhibits the activation of Akt via phosphorylation and the synthesis of cyclin D1 in colon CSCs. **31** effectively suppressed PI-3K activity in vivo, resulting in decreased tumor formation, enhanced identification of cleaved caspase 3, and elevated production of the inflammatory chemokine CXCL8 [94].

### 2.7. Small-Molecule Compounds Targeting Sirtuin Signaling Pathway

Sirtuins are crucial in regulating multiple metabolic processes and are classified as members of the histone deacetylase family III [182,183]. There are seven members called sirtuins (SIRT1-7) [184]. Individual sirtuin members may have different subcellular locations, different expression patterns, and distinct substrates [185]. Sirtuins regulate nonhistone proteins through lysine deacetylation [185]. Recent research has shown that sirtuins are the master regulators of a wide variety of cellular processes, including apoptosis, the cell cycle, extracellular matrix, differentiation, gene expression, DNA repair, proliferation, telomere activity, metabolism, and senescence [186,187]. Several cellular functions mentioned above play crucial roles in maintaining and regulating both normal SCs and CSCs. The recent proposal of inhibiting sirtuins has shown promise as an effective technique for combating cancer. Chemical modifications applied to sirtinol resulted in a collection of SIRT1/2 inhibitors, some of which are more effective than sirtinol itself. The primary objective of these inhibitors is to specifically target SIRT1. Salermide (**32**, Figure 3) had notable effectiveness against colorectal carcinoma CSCs during the testing process, but the SIRT2-selective inhibitor AGK-2 (**33**, Figure 3) had the most powerful effect on glioblastoma multiforme CSCs [95]. Benzodeazaoxaflavins (**34**, **35**, and **36**, Figure 3) are inhibitors of SIRT1/2. They have been found to have pro-apoptotic properties in human U937 leukemia cells. Additionally, when combined with the SIRT1-selective MC2141 (**37**, Figure 3), they have shown antiproliferative effects in CSCs from patients with colorectal carcinoma and glioblastoma multiforme. These CSCs are known to be highly tumorigenic, resistant to conventional cancer chemotherapy, and are responsible, at least in part, for cancer relapse or recurrence [96].

### 2.8. Small-Molecule Compounds Targeting ALDH Signaling Pathway

Aldehyde dehydrogenase 1 (ALDH1) is one of the most extensively studied markers used to select human CSCs. ALDH1 is composed of detoxifying enzymes that catalyze the oxidation of (retina)aldehydes to retinoids [188]. The human mammary cells that were chosen for their higher ALDH1 activity had the most extensive ability to differentiate into various lineages and had the best capability for development in a xenograft model. This suggests that the population of ALDH1 positive cells is enriched with mammary stem cells. Moreover, it was demonstrated that the ALDH1 positive population exhibited greater tumorigenic ability across successive passages, as opposed to the ALDH1 negative population [189]. The precise role of ALDH1 in (mammary) stem cells is not well understood; however, it is believed to be involved in cellular differentiation, primarily through the retinoid signaling pathway [190]. Flow cytometry with fluorescent substrates for ALDH1 has been the established method for assessing ALDH1 activity in live cells, considered the “gold standard” [189,191,192].

The specific isoform of ALDH1A that is responsible for the enzymatic activity is still a subject of debate; nonetheless, ALDH1A1 is often regarded as having the most significant impact. Consequently, extensive research has been conducted on the correlation between the expression of ALDH1A1 protein and clinicopathologic markers. In the majority of tumor types, including colorectal carcinoma [193], clear cell renal cell carcinoma [194], esophageal squamous cell carcinoma [195], gastric cancer [196], urothelial carcinomas of the urinary bladder [197], and squamous cell carcinoma of the head and neck [198], increased ALDH1A1 protein expression was correlated with tumor metastasis and an unfavorable prognosis. The prognostic significance of ALDH1A1 for breast cancer continues to be a subject of controversy, notwithstanding the existence of numerous independent investigations [189,199,200]. A recent meta-analysis identified ALDH1A1 as a biomarker for predicting tumor growth and poor survival in breast cancer patients [201]. Given the existence of high-quality commercial antibodies targeting ALDH1A1, it is crucial to utilize immunohistochemistry to analyze ALDH1A1 expression.

ALDH participates in aldehyde detoxification and retinoic acid synthesis, which contribute to tissue and cellular homeostasis [202,203]. ALDH performs its function in cell differentiation using retinoic acid. ALDH is present in progenitor cells, SCs, and CSCs, making it useful for isolating cell populations with CSC traits from various tumor types [204]. Specific inhibitors demonstrate a decrease in ALDH activity, resulting in reduced cell proliferation, invasion, drug sensitivity, and loss of stem cell characteristics [205,206]. Therefore, ALDH is a promising therapeutic target for treating CSCs [205]. Compound **38** (Figure 3) was discovered to be a unique inhibitor of ALDH1A, demonstrating selectivity over the similar ALDH2 class. The injection of **38** resulted in the depletion of the CD133+ putative CSC population. Additionally, it had a synergistic effect with cisplatin and obtained effective quantities when IP in vivo [97]. All-trans retinoic acid suppresses the stemness facilitated by ALDH-1 and hinders the development and expansion of ovarian tumors [207] and gastric carcinoma [208].

### 2.9. Small-Molecule Compounds Targeting MDM2

Murine double minute 2 (MDM2) plays a crucial role in suppressing the function of p53 by facilitating its ubiquitination and subsequent destruction [209,210,211]. p53 additionally enhances the expression of MDM2, leading to a feedback loop of negative self-regulation [212]. Recent research indicates that p53 plays a role in both non-malignant stem cells and CSCs, and it also has an impact on the microenvironment and CSC niche [213,214]. Consequently, therapeutic approaches have been devised to specifically target CSCs by restoring the normal activity of wild-type p53 (wtp53). These tactics involve the use of RING finger E3 ligases and CSC maintenance. Most inhibitors that target MDM2 are created and formulated to bind competitively to the deep hydrophobic p53-binding cleft of MDM2. This binding prevents the connection between MDM2 and p53, resulting in the activation of p53-mediated cell death and tumor suppression [215,216]. MDM2 overexpression can hinder the functionality of the p53 protein, which is commonly referred to as the “genome guardian.” Glioblastoma stem cells exhibit a high vulnerability to AMG232 (**39**, Figure 3). Out of all the stem cells, the ones with MDM2 gene amplification, specifically the 464T stem cells, were the most responsive to **39**. The concentration of **39** required to inhibit 50% of the growth of these cells, known as the IC_50_, was 5.3 nM. **39** efficiently suppresses the activity of Nestin and ZEB1, which are factors associated with stemness [98]. A recent discovery revealed that MDM2-selective inhibitors stimulate significant expression of MDM4, its homologue. Compound **40** (Figure 3) exhibited a low nanomolar IC50 for both MDM2 and MDM4 targets. Significantly, **40** had the ability to suppress cell proliferation in a manner that depended on the concentration. It achieved an IC_50_ value of 356 ± 21 nM in neuroblastoma SHSY5Y cells, indicating its potency. Furthermore, it effectively hindered the growth of CSC [99].

### 2.10. Small-Molecule Compounds Targeting ROS Signaling

CSCs have reduced levels of intracellular ROS compared to non-CSCs [217,218]. Furthermore, the uncontrolled accumulation of ROS plays a role in the transformation of healthy hematopoietic stem cells into leukemic cells [219,220]. The presence of reduced levels of basal and radiation-induced ROS was seen in CD44+/CD24- breast CSCs, and this was found to be associated with their tumorigenic properties [217]. CD13, also known as Aminopeptidase N, is an enzyme that plays a role in the metabolic route of ROS [221,222], and was essential in the continued existence of CSCs [223]. Dong et al. detected a metabolic shift to glucose metabolism in basal-like breast cancer, accompanied by reduced levels of ROS [224]. They noted that the absence of fructose-1,6-biphosphatase (FBP1) triggers glycolysis, enhances glucose absorption, promotes the synthesis of tetrameric PKM2 macromolecules, and sustains ATP generation under low oxygen conditions. Disruption of FBP1 function also hinders the generation of ROS and the consumption of oxygen. This alteration in metabolism results in an enhanced CSC-like characteristic by enhancing the interaction between T-cell factor and β-catenin.

An imbalance in levels of ROS can result in the proliferation of cells with aberrant growth and the propensity to develop tumors. Hence, maintaining an equilibrium in the quantity of ROS is crucial for both the generation and breakdown processes, which play a significant role in tumor growth and the self-renewal of CSCs. The Glutathione peroxidases (GPx) family is a component of the cellular defense system against ROS and may have a role in regulating cellular oxidative stress and responses mediated by redox signaling. Certain members of the GPx family may also be involved in the self-renewal of CSCs [225,226,227]. Compounds **41** and **42** (Figure 3), two oleanolic acid derivatives, showed inhibition of different types of CSCs. After treatment with both compounds, ROS levels will increase significantly in cancer cells, which may eliminate CSCs. **41** and **42** show potential as anti-CSC drugs and can be further explored as a novel category of chemotherapeutic agents [100]. (-)-15-Methylene-eburnamonine (**43**, Figure 3) eradicates leukemia stem cells. The medicines also reduce the frequency of primary leukemia stem cells in vitro. The cytotoxic effects seem to be caused by the oxidative stress pathways [101].

### 2.11. Small-Molecule Compounds Targeting Other Signaling Pathway

**Figure 4:**
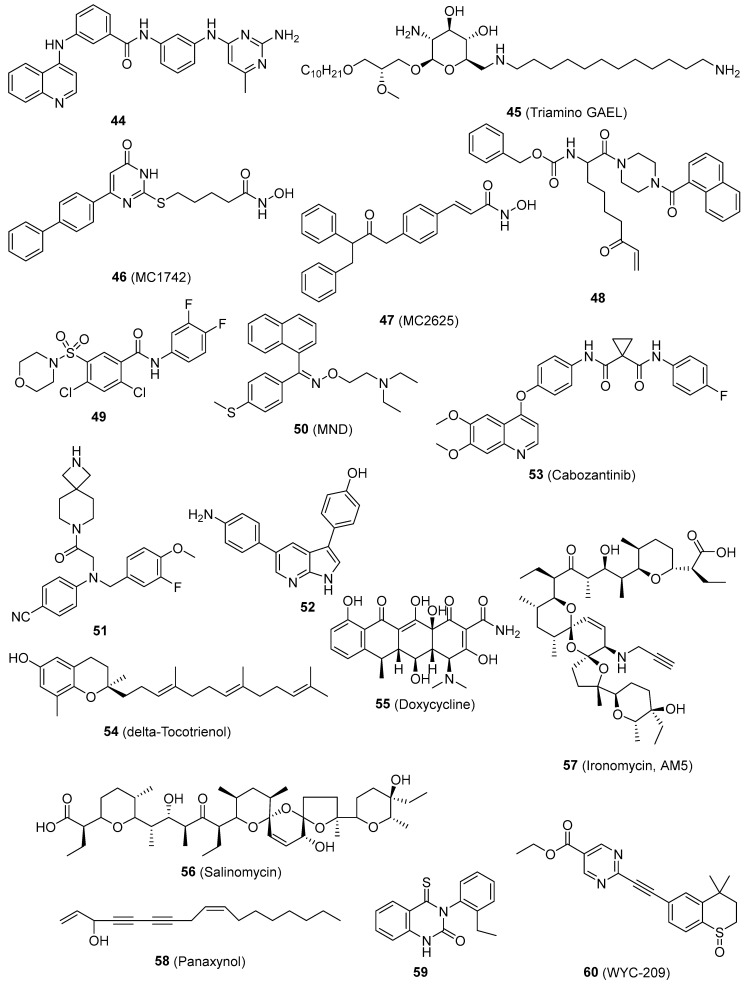
Chemical structures of targeted small-molecule compounds of cancer stem cells.

The DNA methyltransferases (DNMTs) play a role in regulating gene expression through epigenetic mechanisms and are significant targets for cancer treatment. Compound **44** (Figure 4), the initial non-nucleoside DNMTi examined in a CSC line, suppressed cell proliferation in mouse medulloblastoma stem cells [102]. Triamino Glycosylated antitumor ether lipid (Triamino GAEL) (**45**, Figure 4) has more potency than salinomycin (56, Figure 4) against BT474 CSCs. Comprehending the amalgamation of elements that are vital for the increased ability of GAELs to kill cells is significant in order to explore new possibilities for creating powerful substances that can target drug-resistant cancer cells and CSCs [103]. Histone deacetylase (HDAC) inhibitors have been suggested as a potential treatment for counteracting CSCs in solid tumors. When examined in human osteosarcoma, rhabdomyosarcoma, and Ewing’s sarcoma stem cells. MC1742 and MC2625 (**46** and **47**, Figure 4), two HDAC inhibitors (HDACi), increased the levels of acetyl-H3 and acetyl-tubulin and inhibited the proliferation of CSC via inducing death. MC1742 induced osteogenic differentiation at non-toxic dosages [104]. Human tissue transglutaminase (hTG2) is a versatile enzyme with multiple functions. Several clinical disorders, such as CSC survival and metastatic characteristics, are linked to the excessive expression and dysregulation of hTG2 activities. The inhibitor VA4 (**48**, Figure 3), which has been optimized, effectively inhibits the invasion of epidermal CSCs with an EC_50_ value of 3.9 μM [105]. Compound **49** (Figure 4), the most powerful ATX inhibitor, effectively decreased the in vitro chemotherapeutic resistance of 4T1 breast cancer stem-like cells to paclitaxel and greatly reduced B16 melanoma metastasis in vivo [106]. The induction of apoptosis by MND (**50**, Figure 4) was as strong as salinomycin, and it effectively prevented migration, invasion, and the formation of cancer stem cell populations. Furthermore, MND displayed successful regression of tumors in mouse MCF-7 xenografts when administered orally. Investigations into the mechanisms showed that MND significantly inhibited the EGF-induced growth, movement, and tyrosine kinase (TK) signaling in breast cancer cells. Pertussis toxin has the ability to nullify the biological effects of MND [107]. Blocking the activity of lysine specific demethylase 1 (LSD1) has been demonstrated to trigger the maturation of leukemia stem cells in acute myeloid leukemia (AML). Compound **51** (Figure 4) has significant efficacy, as indicated by a Kd value of 32 nM and an EC_50_ value of 0.67 μM in a surrogate cellular biomarker experiment [108]. The DYRK family comprises kinases that are overexpressed in malignancy and regulate multiple cancer hallmarks. The most powerful suppressant, **52** (Figure 4) (with an IC_50_ of 50 nM or less), greatly reduced the viability, clonogenic survival, migration, and invasion of glioblastoma cells with stem cell-like characteristics. Target engagement was verified with genetic knockdown and cellular thermal shift tests. The thermal stability of DYRK1A in cells is enhanced following compound treatment, hence validating its binding in cells [109]. Resveratrol hinders the features of pancreatic CSC in both humans and KrasG12D transgenic mice by blocking the activity of proteins that maintain pluripotency and EMT [68]. Resveratrol hinders the features of pancreatic CSC in both humans and KrasG12D transgenic mice by blocking pluripotency maintenance factors and EMT. A separate investigation on cancer stem-like cells (CD24−/CD44+/ESA+) showed that resveratrol controls FAS expression, hinders lipogenesis, and triggers death in these cells. This represents a new and unique anti-tumor action of resveratrol [69]. Cabozantinib (**53**, Figure 4), a novel therapeutic candidate now undergoing phase II clinical trials, exerts its effects by targeting the surface marker of pancreatic CSC and the tyrosine kinase receptor c-Met. **53** caused a decrease in cancer stem cell markers and the pluripotency transcription factor SOX2, as well as triggered apoptosis in subclones that were treated with gemcitabine for a long period of time [110]. δ-Tocotrienol (VEDT) (**54**, Figure 4), a naturally occurring variant of vitamin E, specifically suppresses the viability, survival, self-renewal, and expression of Oct4 and Sox2 transcription factors in pancreatic ductal adenocarcinoma (PDAC) stem-like cells. VEDT effectively inhibited the proliferation and spread of gemcitabine-resistant PDAC stem-like cells in a model where these cells were transplanted into the appropriate anatomical location [111]. Doxycycline (**55**, Figure 4) has the ability to hinder the survival and reproduction of breast CSCs. Following the administration of **55**, the levels of stem cell factors Oct4, Sox2, Nanog, and CD44 were notably reduced, indicating its potential effectiveness as a therapeutic agent against cancer [112]. Salinomycin (**56**, Figure 4) significantly decreases the percentage of CSCs by more than 100 times compared to paclitaxel. Administering **56** to mice suppresses the growth of mammary tumors in vivo and promotes enhanced epithelial differentiation of tumor cells. Furthermore, the administration of **56** leads to the suppression of breast CSC gene expression [113,114]. Ironomycin (AM5) (**57**, Figure 4), a synthetic derivative of salinomycin (**56**), has enhanced efficacy and specificity against breast CSCs both in laboratory settings and in living organisms. This is achieved by effectively collecting and isolating iron within lysosomes. These findings demonstrate the widespread occurrence of iron balance in breast CSCs, indicating that iron and iron-related processes could be prospective targets for combating these cells [115]. Panaxynol (**58**, Figure 4), a naturally occurring inhibitor of Hsp90, effectively suppressed the capacity of non-small cell lung cancer (NSCLC) CSCs to form spheres at extremely low doses in the nanomolar range. **58** exerted its effects on both the N-terminal and C-terminal regions of Hsp90 through a specific mechanism, while causing little deleterious effects [116]. The coadministration of a thioxodihydroquinazolinone derivative (**59**, Figure 4) and cisplatin exhibits the ability to eradicate cancer stem cell-like populations in ovarian cancer. Continued advancement of thioxodihydroquinazolinone compounds has the potential to result in a more potent therapy for metastatic ovarian cancer that is resistant to cisplatin [117]. WYC-209 (**6**, Figure 4), a man-made retinoid, effectively prevents the growth of malignant murine melanoma tumor-repopulating cells (TRCs, cells similar to CSCs) by 50% at a concentration of 0.19 µM, and this effect is directly proportional to the dosage. WYC-209 predominantly triggers apoptosis of TRCs through the caspase 3 pathway [118].

## 3. Conclusions and Future Prospects

A detailed overview of the small-molecular inhibitors that selectively target several pathways directly linked to the regeneration and stemness of CSCs has been provided. The pathways include Notch, hedgehog, Wnt/β-catenin, NF-κB, STAT3, PI-3K/Akt/mTOR, sirtuin, ALDH, MDM2, ROS, and others. The hypothesis suggests that the occurrence of cancer is attributed to the disturbance of self-renewal mechanisms in stem cells. This suggests that the components of these pathways could be potential targets for therapeutic development. Recent studies indicate that it may be imperative to focus on CSC subpopulations in order to eradicate the whole cancer cell population. Small-molecule compounds are largely acquired for small-molecule discovery through high-throughput screening of compound libraries, natural products, medication repurposing, and structural optimization based on structure-activity connections. From a mechanistic standpoint, a significant majority of small-molecule drugs are categorized as competitive inhibitors, which largely impede their function by reducing the interaction between regulators and substrates. Small-molecule drugs exhibit excellent selectivity, resulting in minimal detrimental effects on normal cells. These molecular chemicals present a different method for treating cancer and give a fresh opportunity for tackling drug-resistant cancers that do not react to conventional therapies. Additionally, it can be utilized in conjunction with other anti-neoplastic medications to augment effectiveness, surmount resistance, and mitigate bad events. The exploration and advancement of small-molecule medicines that specifically target CSCs are slowly becoming recognized as highly effective ways for combating tumors. While numerous small-molecule drugs have demonstrated promising inhibitory actions, there are pressing challenges that require immediate attention. Some small-molecule drugs still need to be improved in terms of their intracellular action because of the selectivity of cell absorption. Some studies have focused solely on investigating the inhibitory effects of small compounds. However, there is still a need to optimize their ADME qualities, such as lipophilicity and solubility. Furthermore, because to the extensive range of subtyping stages and distinct characteristics observed at various stages of cancer and CSCs, it is imperative to provide additional clarification and subdivision regarding the precise suitability of small-molecule compounds. It is crucial to create compound libraries of excellent quality that come from a wide range of sources. The use of Artificial Intelligence and Machine Learning technologies, specifically the DEREPLICATOR+ and DP4-AI tools based on mass spectrometry and NMR, enables quick identification and clarification of chemical structures from intricate materials. Through ongoing study, these revolutionary therapeutic methods are expected to be implemented in clinical settings, potentially resulting in significant advancements in cancer therapy. Ultimately, the ability of small molecules to be used as drugs can be continuously improved, and the characteristics of compounds can be thoroughly examined from several angles, such as pharmacodynamics, pharmacokinetics, and toxicity. As our comprehension of CSC deepens, the discovery of therapeutic drugs specifically targeting CSCs may result in significant advancements in cancer therapies.

To summarize, there has been significant research on CSCs in the field of cancer biology. There is a rising interest in using small-molecule compounds that specifically target CSCs as potential therapies to combat tumors. This article presents a thorough summary of recent progress in important small-molecule compounds and predicts the development of even more effective and specific compounds in the future, thereby providing new possibilities for cancer treatment.

## Figures and Tables

**Table 1:** Targeted small-molecule compounds of cancer stem cells.

Compounds	Mode of Action/Targets	Cancer Types	References
**1** (Epigallocatechin-3-gallate, EGCG)	Notch, Wnt/β-catenin, STAT3, NF-κB	HNSC, lung, breast	[56,57,58]
**2** (GSI-18)	Notch	Brain	[59]
**3** (Vitamin D)	Notch, NF-κB	TNBC	[60]
**4** (Niclosamide)	Wnt, Notch, SHH	Breast	[61]
**5** (Trifluoperazine)	Wnt/β-catenin	Lung	[62]
**6** (All-trans-retinoic acid, ATRA)	Wnt/β-catenin, ALDH1,	HNSC, thyoid, breast, ovarian, gastric carcinoma	[63,64,65]
**7** (Sulforaphane)	Wnt/β-catenin	Breast	[66]
**8** (Resveratrol)	Wnt/β-catenin, pluripotency factor, EMT, FAS	Breast, pancreatic	[67,68,69]
**9** (Curcumin)	Wnt/β-catenin, EMT, SHH, STAT3, NF-κB	Breast, lung, bladder	[58,70,71,72]
**10** and **11** (VS-4718 and VS-6063)	Wnt/β-catenin, FAK	TNBC	[73]
**12** (Ginsenoside Rb1)	Wnt/β-catenin	Ovarian carcinoma	[74]
**13** (Diallyl trisulfide, DATS)	Wnt/β-catenin	Breast	[75]
**14** (Calcitriol)	Wnt/β-catenin	Ovarian	[76]
**15** (Rimonabant)	Wnt/β-catenin	Colon	[77]
**16** (NVP-LDE-225)	SHH	Prostate	[78]
**17** (Cyclopamine)	SHH/Gli 1	Gliomas	[79]
**18** (Vismodegib, GDC-0499)	SHH	Pancreatic	[80]
**19** (Caffeic acid phenethyl ester, CAPE)	NF-κB	Breast	[81]
**20** (Morusin)	NF-κB,	Cervical	[82]
**21** (Disulfiram)	NF-κB, ROS,	Breast	[83]
**22** (Eugenol)	NF-κB,	TNBC	[84]
**23** (Napabucasin, BBI608)	STAT3	Colorectal, gastric/GEJ	[8,85,86,87]
**24** (Galiellalactone)	STAT3, ALDH,	Prostate	[88]
**25** (Honokiol, HNK)	STAT3,	Breast	[89]
**26** (Metformin)	STAT3, NF-κB,	Breast, melanoma	[90]
**27** (Perifosine)	PI-3K/Akt/m-TOR	Breast	[52]
**28** (Sirolimus, Rapamycin)	PI-3K/Akt/m-TOR	Colorectal	[91]
**29** (Rottlerin, ROT)	PI-3K/Akt/m-TOR	Pancreatic,	[92]
**30** (VS-5584)	PI-3K/Akt/m-TOR	Breast, ovarian	[93]
**31** (LY294002)	PI-3K/Akt/m-TOR	Colon	[94]
**32** (Salermide)	SIRT1/2	Colorectal	[95]
**33** (AGK-2)	SIRT2	Glioblastoma multiforme	[95]
**34**, **35**, and **36** (Benzodeazaoxaflavins)	SIRT1/2	Leukemia, colorectal, glioblastoma multiforme	[96]
**37** (MC2141)	SIRT1	colorectal, glioblastoma multiforme	[96]
**38**	ALDH1	Ovarian	[97]
**39** (AMG232)	MDM2	Glioblastoma	[98]
**40**	MDM2	Neuroblastoma	[99]
**41** and **42**	ROS	Breast, melanoma, pancreatic, lung	[100]
**43** ((-)-15-Methylene-eburnamonine)	ROS	Leukemia	[101]
**44**	DNMTs	Medulloblastoma	[102]
**45** (Triamino GAEL)		TNBC, brain, ovarian	[103]
**46** and **47** (MC1742 and MC2625)	HDAC	Osteosarcoma, rhabdomyosarcoma, Ewing’s sarcoma	[104]
**48** (VA4)	hTG2	Epidermal cancer	[105]
**49**	ATX	Breast cancer, melanoma	[106]
**50** (MND)	EGF	Breast	[107]
**51**	LSD1	Acute myeloid leukemia	[108]
**52**	DYPK	Glioblastoma	[109]
**53** (Cabozantinib)	c-Met	Pancreatic	[110]
**54** (δ-Tocotrienol)	EMT	Pancreatic ductal adenocarcinomas	[111]
**55** (Doxycycline)	EMT	Breast	[112]
**56** (Salinomycin)	Iron	Breast	[113,114,115]
**57** (Ironomycin, AM5)	Iron and iron-mediated processes	Breast	[115]
**58** (Panaxynol)	Hsp90	NSCLC	[116]
**59**	ALDH	Ovarian	[117]
**60** (WYC-209)	Caspase 3	Melanoma	[118]

## Abbreviations

ALDH1	Aldehyde dehydrogenase 1
ATRA	All-trans retinoic acid
BCSCs	Breast CSCs
CAPE	Caffeic acid phenethyl ester
COX	Cyclo-oxygenase
CSCs	Cancer stem cells
DATS	Diallyl trisulfide
Dhh	Desert hedgehog
DLL1	Delta-like 1
DNMTs	DNA methyltransferases
EGCG	Epigallocatechin-3-gallate
EGF	Epidermal growth factor
EMT	Epithelial–mesenchymal transition
Epo	Erythropoietin
EpoR	Erythropoietin receptor
FAK	Focal adhesion kinase
FBP1	Fructose-1,6-biphosphatase
GPx	Glutathione peroxidases
HDAC	Histone deacetylase
HDACi	HDAC inhibitors
HNK	Honokiol
HNSC	Head-neck squamous carcinoma
hTG2	Human tissue transglutaminase
Ihh	Indian hedgehog
LPA	Lysophosphatidic acid
LPS	Lipopolysaccharides
LSD1	Lysine-specific demethylase 1
MDM2	Murine double minute 2
NSCLC	Non-small cell lung cancer
PDAC	Pancreatic ductal adenocarcinomas
PKC-δ	Protein kinase C-delta
PI3K	Phosphatidylinositol-3-kinase
ROS	Reactive oxygen species
ROT	Rottlerin
SCs	Stem cells
Shh	Sonic hedgehog
SFN	Sulforaphane
SLNs	Solid matrix of lipidic nanoparticles
TK	Tyrosine kinase
TNBC	Triple-negative breast cancer
